# Long great saphenous vein grafting as temporary coronary bypass for extended left hepatectomy: report of a case

**DOI:** 10.1186/s40792-015-0017-5

**Published:** 2015-01-29

**Authors:** Suefumi Aosasa, Akifumi Kimura, Makoto Nishikawa, Takuji Noro, Hironori Tsujimoto, Kazuo Hase, Junji Yamamoto

**Affiliations:** Department of Surgery, National Defense Medical College, 3-2 Namiki, Tokorozawa, Saitama 359-8513 Japan

**Keywords:** Hepatectomy, Right gastroepiploic artery graft, Coronary artery bypass grafting, Hepatocellular carcinoma

## Abstract

The right gastroepiploic artery (RGEA) has been used in coronary artery bypass grafting (CABG) as an alternative graft. In particular abdominal surgeries, surgery is required to rescue the graft flow into the coronary artery. A 77-year-old male with a history of CABG using RGEA was admitted with a diagnosis of a large hepatocellular carcinoma (HCC) occupying the whole caudate lobe. Preoperative coronary angiography indicated that the graft from the right internal mammary artery to the proximal left circumflex artery was obliterated among three branch bypasses. Following laparotomy, a great saphenous vein was harvested and delivered from the right axial artery to the RGEA graft over the thoracic wall, and the RGEA graft was ligated and divided. Subsequently, extended left hepatectomy was safely performed. Following hepatectomy, the RGEA graft was restored to the former condition, and the temporary graft was removed. After overcoming hyperbilirubinemia, the patient was discharged on postoperative day 28. This experience indicates that temporary bypass using the long great saphenous vein is effective and safe during long and invasive surgeries.

## Background

The right gastroepiploic artery (RGEA) has been used in coronary artery bypass grafting (CABG) as an alternative graft [1,2]. An increased incidence of gastric cancer after CABG using the RGEA has been reported. Performing total gastrectomy with preservation of the RGEA graft has been reported to be feasible, albeit difficult, as careful epigastric manipulation is required to prevent RGEA injury [3-5]. Meanwhile, in particular abdominal surgeries, such as hepatectomy and pancreatoduodenectomy, in which preserving the functioning RGEA graft is unachievable, surgery is required to rescue the graft flow into the coronary artery.

We herein report a case of successful extended left hepatectomy of hepatocellular carcinoma (HCC) using a temporary long great saphenous vein graft to preserve the RGEA graft for CABG.

## Case presentation

A 77-year-old male was admitted to our hospital with a diagnosis of a large liver tumor occupying the whole caudate lobe. He had suffered from weight loss of 5 kg and moderate back pain for the previous 3 months. His upper abdomen was elevated, and a firm, fixed tumor measuring approximately 15 cm in diameter was palpable. The laboratory findings were as follows: hematocrit = 35.8%, platelets = 252 × 10^3^/μl, aspartate aminotransferase (AST) = 51 U/l (normal <35), alanine aminotransferase (ALT) = 13 U (normal <28), bilirubin = 0.6 mg/dl (normal <1.0), albumin = 3.2 g/dl, and prothrombin time = 97.8%. The indocyanine green retention rate after 15 min (ICG-R15) was 26.8%. Screening for hepatitis B and C was negative. The serum concentration of alpha-fetoprotein was 308,900 ng/ml (normal <15), while that of des-gamma-carboxy prothrombin (PIVKA-II) was 20,046 mAU/ml (normal <40).

Eleven years earlier, he had undergone triple bypass grafting with the left internal mammary artery used as a graft to the left anterior descending artery, the right internal mammary artery as a graft to the proximal left circumflex artery, and the RGEA as an *in situ* graft to the posterior interventricular branch of the left circumflex artery via the antegastric and antehepatic routes through an anterior diaphragmatic window. The patient had diabetes mellitus and hypertension, for which he had been taking medication.

**Figure 1:**
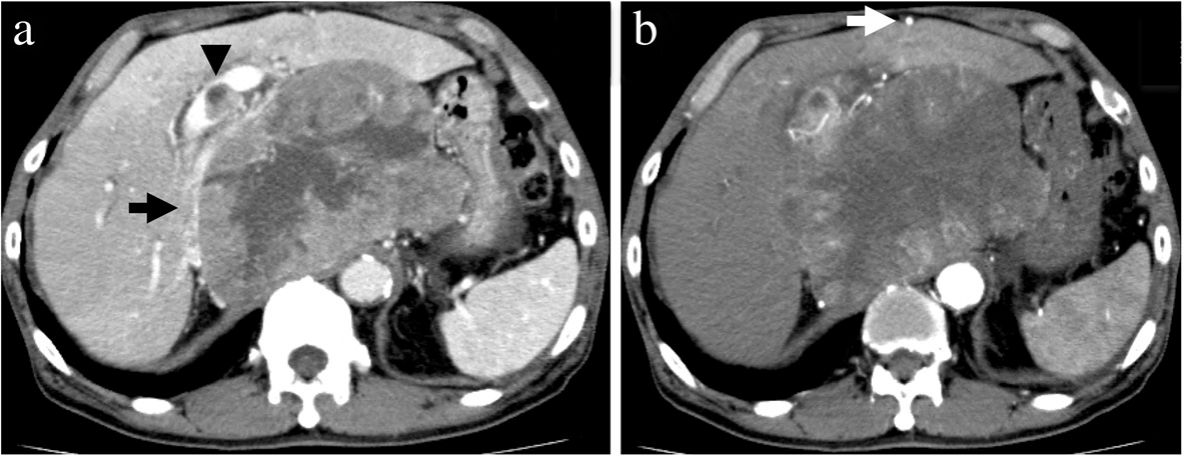
**Enhanced abdominal computed tomography results. (a)** Enhanced abdominal computed tomography (CT) showed a large solitary tumor measuring 15 cm in diameter occupying the whole caudate lobe, subsequently pushing up the inferior vena cava (IVC) ventrally (arrow). In addition, a tumor thrombus (2 cm in diameter) was found at the bifurcation of the portal vein (arrowhead). **(b)** Enhanced CT showed that the right gastroepiploic artery graft passed through the antehepatic route (arrow).

**Figure 2:**
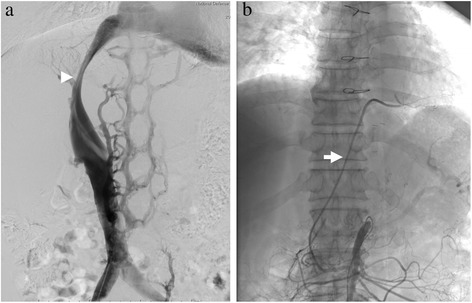
**Preoperative cavography and angiography results. (a)** Preoperative cavography showed the inferior vena cava (IVC) compressed and narrowed by the large tumor (arrowhead). **(b)** Preoperative angiography showed that the patent right gastroepiploic artery graft branched from the superior mesenteric artery (arrow).

Preoperative enhanced abdominal computed tomography (CT) showed a large solitary tumor measuring 15 cm in diameter occupying the whole caudate lobe, subsequently pushing up the inferior vena cava (IVC) ventrally and extending to the right anterior segment sticking to the confluence of the right hepatic vein (RHV). A tumor thrombus (2 cm in diameter) was found at the bifurcation of the portal vein, and the RGEA graft passed through the antehepatic route (Figure 1). The IVC was found to be compressed and narrowed by the tumor with significant enlargement of the azygos vein system on preoperative cavography. In addition, angiography showed that he had anomaly of the RGEA arose from the superior mesenteric artery directly, and this graft of the RGEA was patent (Figure 2); however, the right internal mammary artery graft was found to be obstructed. Therefore, in order to secure the circulation of the left circumflex artery during hepatectomy, temporary bypass to salvage the function of the RGEA graft using the harvested great saphenous vein was planned. No abnormal findings were noted in any other organs.

**Figure 3:**
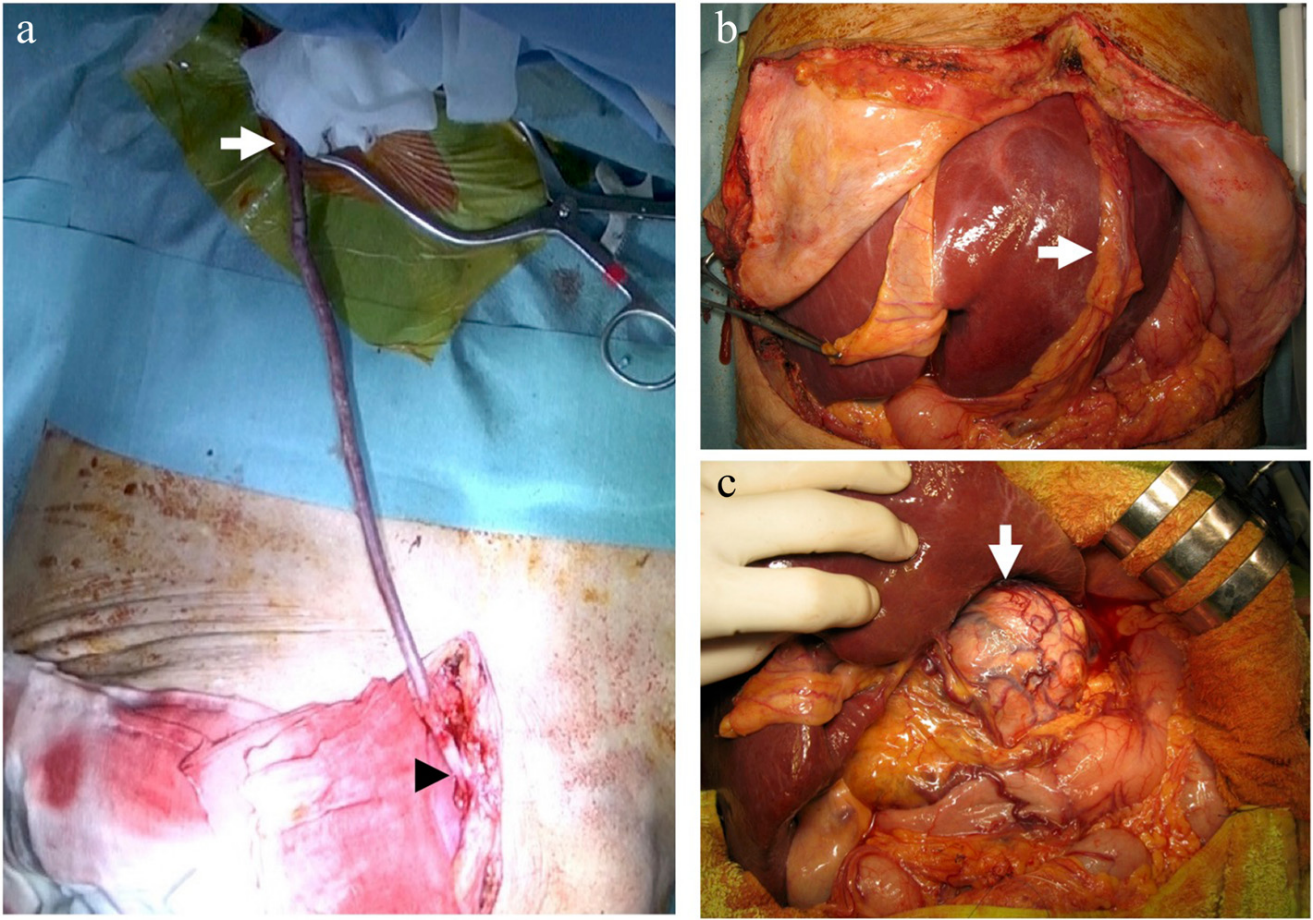
**Intraoperative findings. (a)** A long great saphenous vein graft anastomosed to the axillary artery (arrow) was delivered over the thoracic wall and anastomosed to the right gastroepiploic artery (RGEA) graft (arrowhead). **(b)** The RGEA graft passed through the antehepatic route. **(c)** A large tumor occupying the caudate lobe was observed following the ligation and division of the RGEA graft.

Electrocardiography, the left radial arterial line, a Swan-Ganz catheter (Baxter Healthcare Co., CA, USA) and transesophageal echocardiography were used for monitoring. The abdomen was entered via an inverted T-shaped incision in the upper abdomen, and the RGEA graft on the left lobe of the liver was first identified. There was no ascites or peritoneal metastasis, and the liver showed almost normal findings. A 40-cm-long great saphenous vein graft was harvested from the left leg. End-to-side anastomosis was then performed between the vein graft and axillary artery under a 3-cm right infraclavicular skin incision. The vein graft was delivered over the thoracic wall and anastomosed to the RGEA graft in an end-to-side fashion without occlusion of the flow to posterolateral branch of the circumflex artery (Figure 3A). After confirming a sufficient blood flow from the axillary artery to the posterolateral branch of the circumflex artery via the temporary saphenous vein graft using transient time flow and Doppler velocity measurements (VeriQ3™; MediStim, Oslo, Norway), the RGEA graft was ligated and divided just proximal to the anastomosis (Figure 3B). During these procedures, neither ischemic changes nor hemodynamic deterioration were observed on the monitors.

Elevation of the left lobe was safely performed, and a large tumor located in the caudate lobe was visible (Figure 3C). The tumor strongly adhered to the diaphragm, hepatoduodenal ligament, and retroperitoneum. Intraoperative ultrasonography showed a tumor thrombus at the root of the left portal branch from the caudate lobe. Following cholecystectomy, dissection of the hilar vascular structures was performed with considerable difficulty. After ligating and severing the left hepatic artery, the portal venous trunk and right portal branch were taped, and the tumor thrombus at the portal bifurcation was removed via an incision made on the left portal branch, which was subsequently closed with sutures. Mobilization of the liver was performed from the right, securing the inferior right hepatic vein and two thick short hepatic veins. Then, the supra- and infrahepatic IVC was taped, and Belghiti's maneuver [6] was carried out, securing the aforementioned three hepatic veins. Liver parenchymal transection was then performed using the clamp crushing method [7] up to the right side of the RHV under hepatic vascular exclusion, and the main trunk of the middle and left hepatic veins was divided with an endocutter, after which the RHV was ligated and divided, thus all of three major hepatic vein were divided. Resection was completed by detaching the tumor from the diaphragm. Following removal of the tumor, the RGEA graft was restored to its former condition, and the temporary graft and saphenous vein were removed after confirming a sufficient blood flow in the RGEA graft. This was a very difficult procedure due to the large tumor size, firm adhesion around the tumor, and development of the collateral route. The temporary bypass time was 16 h and 4 min, and the total operative time was 21 h and 34 min. The total amount of blood loss was 7,089 g, and 4,480 ml of red blood cell and 2,400 ml of fresh frozen plasma were transfused. The resected specimen weighed 1,476 g, and the tumor measured 17.1 × 13.2 × 11.5 cm, with a histological diagnosis of poorly differentiated hepatocellular carcinoma extensively invading the portal venous system without fibrosis. No positive surgical margins or metastases to the regional lymph nodes were confirmed microscopically.

The patient had an uneventful postoperative course, except for temporary hyperbilirubinemia (maximum serum total bilirubin = 7.2 mg/dl), and was discharged on postoperative day 28. Three months after the surgery, the serum AFP value decreased to 20 ng/ml. However, 10 months after the surgery, multiple lung-limited metastases were found. When last seen at a follow-up consultation 20 months after the surgery, the patient was found to be doing well under treatment with chemotherapy with sorafenib.

### Discussion

Graft injuries during abdominal surgery after coronary artery bypass grafting using the RGEA may suddenly cause coronary failure and fatal arrhythmia [8]. In order to avoid graft injury, it is important to understand how to harvest and where to place the graft to the CABG. Upper abdominal surgeries, such as gastrectomy, after CABG using the RGEA have been reported to be performed safely with careful harvesting [3-5]. However, in particular procedures, such as pancreatoduodenectomy or hepatectomy, division of the RGEA graft is often unavoidable. In our case, the tumor was too large to be manipulated without removing the RGEA graft, which ran in front of the tumor. In addition, in cases of pancreatoduodenectomy, it is often difficult to preserve the RGEA and remove malignancies [9,10].

As for optional repeated bypass grafts to the coronary artery, moderate to long saphenous veins have been reportedly used [11,12]. Yaryura et al. [12] first reported the use of minimally invasive coronary artery bypass employing a saphenous vein graft with an inflow from the left subclavian artery via the intrapleural route to the diagonal artery in a patient with obstruction of two saphenous vein grafts. Ohtsuka et al. [11] applied this technique to salvage the RGEA graft *in situ* 10 days before pancreatoduodenectomy. They redirected the blood flow from the right axillary artery to the RGEA graft on the liver under thoracoscopic guidance. One problem associated with these techniques is distortion/kinking of the graft due to its length and the existence of particular curves along the vein graft, first at the entry into the chest cavity through the first intercostal space and second at the medial margin of the lung tissue [11]. Furthermore, the long-term durability of the long vein graft with an inflow from the subclavian/axillary artery is unknown. Stripping of the long great saphenous vein is commonly performed to treat varicose veins in the lower extremities, without any long-term morbidities [13].

As for the assessment of the preoperative liver function, the current patient showed a relatively high value for ICG-R15 at 26.5%, which ordinarily indicates a moderately impaired hepatic functional reserve. However, he also demonstrated negative findings for hepatitis viral infection in addition to normal values for the platelet count, prothrombin time, and serum albumin and liver enzyme levels. Based on the patient's Child-Pugh score of Grade A and normal liver function, with the exception of the ICG-R15 value, he was found to have a sufficient hepatic functional reserve, with a calculated remnant liver volume of 58% on CT volumetry. Therefore, we chose to perform hepatectomy versus treatments, including transcatheter arterial chemoembolization or chemotherapy with sorafenib. And from the technical point of view, the tumor occupied the entire caudate lobe and covered the hilar plate, subsequently protruding outwards and occupying the anterior superior segment of the right liver and stretching to the right hepatic vein, thus necessitating extended left hepatectomy with resection of the RHV. Finally, the outflow of the remnant liver was secured by conserving the inferior right hepatic vein and two thick short hepatic veins, diagnosed based on preoperative imaging [14]. Furthermore, (1) the patient had a pressing condition with back pain and body weight loss, (2) one of the three bypasses was obstructed, and (3) restoring the previous GEA graft was rather easy after removing the large liver tumor; therefore, a temporary bypass was used to rescue the graft flow into the coronary artery during hepatectomy.

## Conclusions

Temporal long saphenous vein grafting from the axillary artery via the extrapleural route can be safely used in particular situations during abdominal surgery to salvage the RGEA graft *in situ*.

## Consent

Written informed consent was obtained from the patient for publication of this case report and any accompanying images. A copy of the written consent is available for review by the Editor-in-Chief of this journal.

## Abbreviations

ALT: Alanine aminotransferase
AST: Aspartate aminotransferase
CABG: Coronary artery bypass grafting
CT: Computed tomography
HCC: Hepatocellular carcinoma
ICG-R15: Indocyanine green retention rate after 15 minutes
IVC: Inferior vena cava
PIVKA-II: Des-gamma-carboxy prothrombin
RGEA: Right gastroepiploic artery
RHV: Right hepatic vein
